# Risk factors for perineal trauma in the primiparous population during non-operative vaginal delivery

**DOI:** 10.1007/s00192-019-03944-7

**Published:** 2019-05-02

**Authors:** Joanna C. D’Souza, Ash Monga, Douglas G. Tincello

**Affiliations:** 1 Faculty of Medicine, University of Southampton, University Hospitals Southampton NHS Foundation Trust, Tremona Road, Southampton, SO16 6YD UK; 2 Department of Urogynaecology, University Hospitals Southampton NHS Foundation Trust, Princess Anne Hospital, Coxford Road, Southampton, SO16 5YA UK; 3 Department of Health Sciences, College of Life Sciences, University of Leicester, University Road, Leicester, LE1 7RH UK

**Keywords:** Obstetric anal sphincter injuries (OASIS), Obstetric anal sphincter injury (OASI), Perineal trauma, Primiparity

## Abstract

**Introduction and hypothesis:**

Obstetric anal sphincter injuries (OASIS) are more common in the primiparous population, especially during operative vaginal delivery (OVD). It would therefore be interesting to establish what factors influence the risk of OASIS when adjusting for the risk bias of OVD.

**Methods:**

Retrospective analysis of prospectively collected data from the maternity database at University of Southampton NHS Foundation Trust of primiparous women sustaining OASIS during a singleton, term, cephalic, non-operative vaginal delivery between 2004 and 2015. A control comparison was made of women with otherwise identical birthing conditions but resulting with intact perinea, delivering between 2014 and 2015. Univariate and multivariate logistic regression compared maternal, intrapartum and neonatal factors of those sustaining OASIS with those with an intact perineum.

**Results:**

Seven hundred fifty-six women sustaining an OASI met the criteria, and comparisons were made with a control population of 212. Those sustaining an OASI were significantly older (*p* < 0.001), more likely to be Asian (4.6-fold, *p* < 0.001) and had heavier babies, with a 3.6-fold greater proportion over 4 kg (*p* = 0.001). They were more likely to have delivered post-term and had longer second stages of labour (both *p* < 0.001). Epidural anaesthesia was associated with a 67% reduction in OASIS.

**Conclusions:**

These findings support previous research regarding risk factors for OASIS. By controlling for bias of risk associated with operative vaginal delivery, this revealed a potential protective effect of the use of regional anaesthesia.

## Introduction

Perineal trauma at childbirth is a common occurrence, affecting > 85% of all vaginal deliveries in the UK [1]. The rate of obstetric anal sphincter injuries (OASIS), the most severe form of perineal trauma, tripled during the previous decade and the median UK OASIS rate is 2.85% (0–8%) [2, 3]. Affecting the anal sphincter muscles, these injuries can result in significant morbidity and are a contributing factor to longer-term anal incontinence and faecal urgency [4]. The primiparous population is at 2.3-fold increased risk of OASIS [3]. Increasing maternal age, Asian ethnicity, infantile macrosomia (birthweight > 4 kg) and operative vaginal birth (OVD) (forceps > vacuum) are also risk factors for OASIS [5, 6]. Due to the multifactorial nature of OVD, it is difficult to identify the individual effect which other associated factors have on increasing the risk of OASIS, for instance, use of regional anaesthesia and having a prolonged second stage of labour. Therefore, the aim of this study was to investigate what maternal, intrapartum and neonatal factors make sustaining an OASI more likely when adjusting for OVD, therefore focusing specifically on the risk of OASIS at normal vaginal birth (NVD) in the primparous population.

## Methods

Data from an electronic maternity database were analysed via retrospective analysis of prospectively collected data. The sample included all primiparous women sustaining an OASI from January 2004 to December 2015 during a singleton, term, cephalic, non-operative vaginal delivery at the University of Southampton NHS Foundation Trust. Comparisons were made with a control group of primiparous women who had normal vaginal deliveries resulting in no perineal trauma. Data regarding the control group were extrapolated from the same maternity database; the data search included all primiparous women with a documented ‘intact’ perineum. These women had otherwise identical birthing conditions to those of the study group. These women delivered between January 2014 and December 2015.

Informed consent was not required as data were anonymised and no patient contact was made.

Data were checked for missing input, and women who had multiple, pre-term, non-cephalic and operative deliveries were excluded from the analysis. Data on third- and fourth-degree OASIS were combined. Univariate analysis was carried out comparing maternal, intrapartum and neonatal factors between women sustaining an OASI and the control population. Continuous data were analysed using the Mann-Whitney U test, as the Kolmogorov-Smirnov test determined the distribution to be non-parametric. Categorical data were analysed with the chi-square test. Binary logistic regression was used to calculate the independent odds ratio (OR) of OASIS, including factors reaching statistical significance (*p* < 0.05). Analysis was carried out using IBM SPSS v.24.

Ethical approval was not required for this database study as there was no direct contact with patients.

## Results

In the 12-year time period there were 68,606 births, of which 52,412 were singleton, term, cephalic, vaginal deliveries. Of these, 41.2% (21,605/52,412) were to primiparous women. The overall prevalence of OASIS was 3.5% (1841/52,412). Just over two thirds (68.9%) of all OASIS were sustained by primiparous women at a rate of 5.9% (1269/21,605), which was 3.1-fold greater than the Trust’s contemporaneous multiparous rate [5.9% vs. 1.9% (572/30,807), difference 4.0% (95% CI 3.7, 4.3)]. These figures included all modes of vaginal delivery.

Table 1 shows univariate analysis of maternal, intrapartum and neonatal factors comparing those sustaining OASIS (*n* = 756) at normal vaginal delivery (NVD) with the control sample (*n* = 212). Women sustaining OASIS were significantly older (median age 28 vs. 24, *p* < 0.001) and had achieved a higher level of education (43.8% graduates from university vs. 24.4%, *p* < 0.001). Significant differences were seen in the frequency of OASIS among women of non-Caucasian ethnicity, namely there were 4.9-fold more Asian women sustaining an OASI (14.6% vs. 3.0%*p* < 0.001).

**Table 1:** Univariate analysis comparing those sustaining an OASI with the control population

		**Women sustaining OASIS** (n=756)	**Control group** (n=212)	**p-value**
**Age**	Median	28 (15 – 45)	24 (15 – 40)	**p<0.001**^**a**^
	By age category:			
	<20 20-25 25-30 30-35 35-40 >40	36 (4.8%) 147 (19.4%) 263 (34.8%) 242 (32.0%) 59 (7.8%) 9 (1.2%)	43 (20.3%) 75 (35.4%) 58 (23.4%) 29 (13.7%) 6 (2.8%) 1 (0.5%)	
**Ethnicity** (OASIS n=734, Control n=203)	Caucasian Asian Black	609 (83.0%) 107 (14.6%) 18 (2.5%)	194 (95.6%) 6 (3.0%) 3 (1.5%)	**p<0.001**^**b**^
**Education** (OASIS n=750, Control n=209)	Higher (Graduate) Lower	321 (43.8%) 429 (57.2%)	51 (24.4%) 158 (75.6%)	**p<0.001**^**b**^
**Gestation (>40 weeks)**		437 (57.8%)	94 (44.3%)	**p<0.001**^**b**^
**Induction of labour**		113 (14.9%)	37 (17.5%)	p=0.373^**b**^
**Epidural anaesthesia**		42 (5.6%)	29 (13.7%)	**p<0.001**^**b**^
**Length of 2**^**nd**^**stage (mins)**	Median	62 (2 – 375)	35 (2 – 192)	**p<0.001**^**a**^
**Head position (if OP)** (OASIS n=81 (2014-15 only), Control n=211)		1 (1.2%)	6 (2.8%)	p=0.421^**b**^
**Birth weight (g)**	Median % over 4Kg	3500 (2260 – 4800) 81 (10.7%)	3245 (2020 – 4450) 7 (3.3%)	**p<0.001**^**a**^ **p=0.001**^**b**^

^a^Mann-Whitney U Test, ^b^ Chi-square Test, p≤0.05 (p values in bold type met statistical significance)

Those suffering OASIS had significantly heavier babies [median weight (g) 3500 vs. 3245 *p* < 0.001] with a 3.6-fold greater proportion weighing > 4 kg (10.7% vs. 3.3%, *p* = 0.001). They were more likely to deliver post-term (57.8% vs. 44.3%, *p* < 0.001) and have a longer second stage of labour [median time (min) 62 vs. 35, *p* < 0.001]. The epidural anaesthesia was associated with a decreased chance of sustaining an OASI [5.6% vs. 13.7% (control), *p* < 0.001]. No significant differences were seen when analysing whether the labour was induced or whether the foetal head was malpresented [whether occiput-posterior (OP) or not]. The factors remaining independently associated with the risk of OASIS after binary logistic regression are shown in Table 2. Infantile macrosomia and giving birth post-term were associated with a 3.2- and 1.8-fold increased risk of sustaining a sphincter injury, respectively. When adjusting ethnicity to only include Caucasian and Asian women, those sustaining an OASI were 6.5 times more likely to be of Asian ethnicity (OR 6.553, 95% CI 2.773–15.483, *p* < 0.001). Epidural anaesthesia was associated with a 67% reduction in OASIS.

**Table 2:** Factors independently associated with the risk of an OASI after binary logistic regression

	OR	95% CI	p-value
Maternal age (years)	1.147	1.107 – 1.188	p<0.001
Ethnicity	3.592	1.966 – 6.563	p<0.001
If baby >4Kg (%)	3.201	1.390 – 7.367	p=0.006
Gestation (>40 weeks)	1.832	1.295 – 2.592	p=0.001
Epidural anaesthesia	0.326	0.171 – 0.624	p=0.001
Length of 2^nd^ stage (mins)	1.009	1.004 – 1.014	p<0.001

OASIS group n=729, Control group n=200

## Discussion

### Main findings

This study aimed to assess what maternal, intrapartum and neonatal factors influence the risk of OASIS in the primiparous population during non-operative vaginal childbirth. This was achieved by using a control comparison of primiparous women with a documented ‘intact perineum.’

Although the Trust’s overall OASI rate was slightly higher than the national average (3.5% vs. 2.9%), the primiparous OASI rate was very similar (5.9% vs. 6.1%) [3]. In agreement with previous studies, we found advancing maternal age and Asian ethnicity to be associated with an increased risk of sustaining an OASI [5–8].

In line with previous studies we also found women having an OASI to have larger babies, with a greater proportion > 4 kg [5, 6, 8]. We also discovered a disparity in the portion of women delivering post-term when comparing those sustaining an OASI with the control population, which would also be associated with increased infant size. Prolonged second stage of labour, or rather the resultant effect of prolonged tension on the perineal tissues, increased the risk of sustaining an OASI, even in the absence of OVD [6, 8].

Contrary to other studies, we did not find women delivering a baby in the OP position to be at greater risk of OASI [9, 10]. The use of epidural was associated with a decreased risk of sustaining an OASI and the process of induction or augmentation of labour was not associated with an increased risk of OASIS.

### Strengths and limitations

One of the strengths of this study is that we controlled for the risk-potentiating effect of OVD by excluding women having either forceps or vacuum extractions from the analysis. This also therefore removed any potential bias when analysing factors known to be affected by or associated with OVD, e.g. prolonged second stage, episiotomy or epidural anaesthesia. Previous studies have used vaginal spontaneous delivery as the reference in logistic regression when analysing the effect of OVD, but then have included all modes of delivery in the analysis of other factors. Additionally, other studies have made comparisons between those sustaining OASIS and those sustaining all other degrees of perineal trauma (including intact perineum, first and second degree and episiotomy), whereas our study only included those with an ‘intact perineum’ [3, 5, 6, 8]. This allows for a cleaner ‘all versus nothing’ analysis, removing the potential for bias and inclusion of undiagnosed OASIS into the control group.

A significant limitation of this data-based study was that because of being limited to only analysing the variables recorded in the birth records, we were unable to review specific intrapartum practices and their effect on outcomes. For instance, whether any techniques or measures known to protect the perineum and reduce the risk of OASI were implemented, e.g. manual perineal protection or application of a warm compress during the second stage [11]. We recognise that analysis of such confounders would have resulted in more robust, applicable evidence but this was unfortunately beyond this study’s remit.

### Interpretation

A possible explanation for the increased risk of OASIS with advancing maternal age is a decrease in the elasticity of connective tissues due to loss of function and strength of connective tissues with increasing age [5, 12]. Previous studies have shown those of higher economic status to be associated with an increased risk of perineal trauma [10, 13]. We analysed the academic success and found those with higher educational achievements (university graduates) to be of increased risk. The most likely explanation for the increased risk of OASIS in women of Asian ethnicity is ethnic variation in perineal body length, where Asian women tend to have shorter perineal bodies [14].

We expected our study to be in agreement with previous research revealing women delivering an OP baby to be at greater risk of OASI due to the larger diameter of the presenting part, but no significant difference was seen [9, 10]. As these studies did not adjust for delivery mode, this could possibly be due to a combined effect of head malpresentation and use of instrument increasing the pressure on the perineal tissues rather than malpresentation alone. However, it is worth noting that the information available regarding this variable was limited to just 2 years’ worth of data and hence the population may not have been sufficient to provide any meaningful conclusions. Previous studies have also shown an increased risk of OASIS in induction of labour or augmentation with oxytocin, but when excluding OVD we found no association [15]. Therefore, the injury sustained is more likely to be due to the need for an OVD rather than the initial induction or augmentation processes.

Epidural has been associated with an increased rate of OASIS but this has not previously been adjusted for the mode of vaginal birth [16–18]. We expected epidural anaesthesia would potentiate the risk of OASI because of the association of regional anaesthesia with the prolonging of the second stage and resultant need for an OVD, both known as risk factors for OASIS. Our study showed epidural at NVD to be protective against OASIS. This could be due to better visualisation and support of the perineum by the accoucher due to maternal immobility and effective analgesia leading to better control and ability of the mother to follow instruction regarding pushing at crowning.

In common with previous studies this research has highlighted an ‘at-risk’ population through the identification of certain risk factors which make sustaining an OASI more likely. This emphasises the importance and need for further education of antenatal care providers and patients alike to ensure preventative measures are particularly emphasised for these women, i.e., primiparous women of advanced maternal age, of Asian ethnicity or carrying larger babies. Discussions should be undertaken antenatally with these ‘at-risk’ women to consider the impact that vaginal birth could have on their perinea, and more specifically on their anal sphincters. This would ensure that they are fully informed and engaged in decisions regarding the intrapartum care they receive. These discussions should include the use of preventative measures such as manual perineal protection, a warm perineal compress during the second stage and a low threshold for medio-lateral episiotomy as well as a low threshold for caesarean delivery in the event of a prolonged second stage or when cephalopelvic disproportion is suspected. It is however obvious that the preventative measures should be considered universally and not exclusively in higher risk populations, as all women would benefit from such measures.

## Conclusion

This research is novel as we controlled for bias associated with OVD by focusing purely on primiparous women achieving an NVD. Additionally, we used a control population with documented ‘intact’ perinea. The findings support previous research in recognising increased maternal age, Asian ethnicity, prolonged second stage, post-term delivery and infantile macrosomia as risk factors for OASIS. This study showed a potential protective effect of the use of regional anaesthesia. Better consideration needs to be made in identifying and counselling ‘at-risk’ women in the antenatal period to ensure measures are in place to aid the prevention of perineal trauma.
